# Antagonism of dsRNA-Induced Innate Immune Pathways by NS4a and NS4b Accessory Proteins during MERS Coronavirus Infection

**DOI:** 10.1128/mBio.00319-19

**Published:** 2019-03-26

**Authors:** Courtney E. Comar, Stephen A. Goldstein, Yize Li, Boyd Yount, Ralph S. Baric, Susan R. Weiss

**Affiliations:** a Department of Microbiology, Perelman School of Medicine at the University of Pennsylvania, Philadelphia, Pennsylvania, USA; b Department of Epidemiology, University of North Carolina at Chapel Hill, Chapel Hill, North Carolina, USA; c Department of Microbiology and Immunology, University of North Carolina at Chapel Hill, Chapel Hill, North Carolina, USA; Icahn School of Medicine at Mount Sinai; University of Iowa; Arizona State University

**Keywords:** MERS-CoV, coronavirus, interferon antagonist, viral accessory proteins

## Abstract

Middle East respiratory syndrome coronavirus (MERS-CoV) was first identified in 2012 as a novel etiological agent of severe respiratory disease in humans. As during infection by other viruses, host sensing of viral double-stranded RNA (dsRNA) induces several antiviral pathways. These include interferon (IFN), oligoadenylate synthetase (OAS)-RNase L, and protein kinase R (PKR). Coronaviruses, including MERS-CoV, potently suppress the activation of these pathways, inducing only modest host responses. Our study describes the functions of two accessory proteins unique to MERS-CoV and related viruses, NS4a and NS4b, during infection in human airway epithelium-derived A549 cells. NS4a has been previously characterized as a dsRNA binding protein, while NS4b is a 2′,5′-phosphodiesterase with structural and enzymatic similarity to NS2 encoded by mouse hepatitis virus (MHV). We found that deletion of NS4a results in increased interferon lambda (*IFNL1*) expression, as does mutation of either the catalytic site or nuclear localization sequence of NS4b. All of the mutant viruses we tested exhibited slight decreases in replication. We previously reported that, like MHV NS2, NS4b antagonizes OAS-RNase L, but suppression of IFN is a previously unidentified function for viral phosphodiesterases. Unexpectedly, deletion of NS4a does not result in robust activation of the PKR or OAS-RNase L pathways. Therefore, MERS-CoV likely encodes other proteins that contribute to suppression or evasion of these antiviral innate immune pathways that should be an important focus of future work. This study provides additional insight into the complex interactions between MERS-CoV and the host immune response.

## Abstract (precis)

Middle East respiratory syndrome coronavirus (MERS-CoV) is the second novel zoonotic coronavirus to emerge in the 21st century and cause outbreaks of severe respiratory disease. More than 2,200 cases and 800 deaths have been reported to date, yet there are no licensed vaccines or treatments. Coronaviruses encode unique accessory proteins that are not required for replication but most likely play roles in immune antagonism and/or pathogenesis. Our study describes the functions of MERS-CoV accessory proteins NS4a and NS4b during infection of a human airway-derived cell line. Loss of these accessory proteins during MERS-CoV infection leads to host antiviral activation and modestly attenuates replication. In the case of both NS4a and NS4b, we have identified roles during infection not previously described, yet the lack of robust activation suggests much remains to be learned about the interactions between MERS-CoV and the infected host.

## INTRODUCTION

Middle East respiratory syndrome coronavirus (MERS-CoV) is a recently emerged, highly pathogenic coronavirus first identified in the Middle East in 2012 (1, 2). Following the 2002 to 2003 severe acute respiratory syndrome (SARS)-CoV pandemic, MERS-CoV is the second zoonotic coronavirus discovered in the 21st century. Although cases have been largely concentrated on the Arabian Peninsula, a large travel-associated outbreak in South Korea in 2015 highlights that MERS-CoV remains a global concern. MERS-CoV circulates in dromedary camels in Africa and the Middle East, having established a reservoir in camels, while closely related viruses are found in African bats, suggesting a bat origin for MERS-CoV or its direct ancestors (3–8).

Like all coronaviruses, MERS-CoV has a large positive-sense single-stranded RNA (ssRNA) genome of 30,119 nucleotides in length. The 5′ two-thirds of the genome encodes the functionally conserved replicase proteins, while a core set of structural proteins are encoded by all viruses of the Betacoronavirus genus in the 3′ 10 kb. Additionally found in the 3′ end of the genome are accessory genes specific to each *Betacoronavirus* subgenus, interspersed with structural genes. The MERS-CoV accessory genes are found only in other betacoronaviruses of the subgenus Merbecovirus (formerly lineage C), while betacoronaviruses of other subgenera such as mouse hepatitis virus (MHV) (Embecovirus [lineage A]) and SARS-CoV (Sarbecovirus [lineage B]) carry unique accessory genes.

Several accessory proteins encoded by MHV and SARS-CoV have been identified as antagonists of the innate immune response (9), as have some MERS-CoV accessory proteins (10–14). Several studies utilizing ectopically expressed protein and reporter systems have identified NS4a, NS4b, and NS5 as putative interferon (IFN) antagonists, but these studies may not faithfully recapitulate the complex interactions between viral and host factors present during infection (11, 13, 15–17). More recent studies utilizing recombinant MERS-CoV have more completely elucidated the functions of some of these proteins, but conflicted with early reporter studies. NS4a, a double-stranded RNA (dsRNA) binding protein, prevents the generation of protein kinase R (PKR)-induced stress granules in some cell types (18). We reported previously that NS4b is a homolog of the NS2 protein of MHV and closely related betacoronaviruses of the subgenus *Embecovirus* (formerly lineage A), has 2′,5′-phosphodiesterase (PDE) activity, and acts as an antagonist of the oligoadenylate synthetase (OAS)-RNase L pathway (19). In contrast to the *Embecovirus* PDEs, NS4b has an N-terminal nuclear localization signal (NLS) and is localized primarily to the nucleus of infected cells (16, 19). NS4b has also been reported to antagonize NF-κB nuclear translocation during MERS-CoV (12, 14, 18, 19), as has NS5 (10).

Building on our previous study characterizing NS4b as an OAS-RNase L antagonist (19), we have used recombinant MERS-CoV to further elucidate the roles of NS4a and NS4b during infection of human airway epithelium-derived A549 cells (20). Consistent with earlier studies, NS4a prevents phosphorylation of PKR and the induction of IFN and interferon-stimulated gene (ISG) expression. However, PKR activation in the absence of NS4a does not result in phosphorylation of eIF2α (eukaryotic initiation factor 2α) or translation arrest in A549 cells, in contrast to recent findings in a different cell type (18). Unlike other viral dsRNA binding proteins such as vaccinia virus E3L (21) and influenza virus NS1 (22), NS4a does not play a significant role in OAS-RNase L antagonism during MERS-CoV infection, as deletion of NS4a does not result in RNase L activation or enhance RNase L activation in the context of MERS-CoV encoding catalytically inactive NS4b.

Our studies of NS4b reveal that in addition to antagonizing OAS-RNase L and preventing NF-κB activation, NS4b antagonizes *IFNL1* expression, with this function dependent on both its catalytic activity and nuclear localization and independent of its interaction with the OAS-RNase L pathway. This is a unique role for virus-encoded phosphodiesterases, which otherwise lack an NLS and act solely as OAS-RNase L antagonists (12, 23–26). Together, the results demonstrate that NS4a and NS4b mediate both expected and unexpected functions during MERS-CoV infection and further demonstrate the importance of studying the function of these proteins in the context of infection to uncover the full range of their interactions with the innate immune response.

## RESULTS

### Construction and characterization of recombinant NS4a and NS4b MERS-CoV mutants.

**FIG 1:**
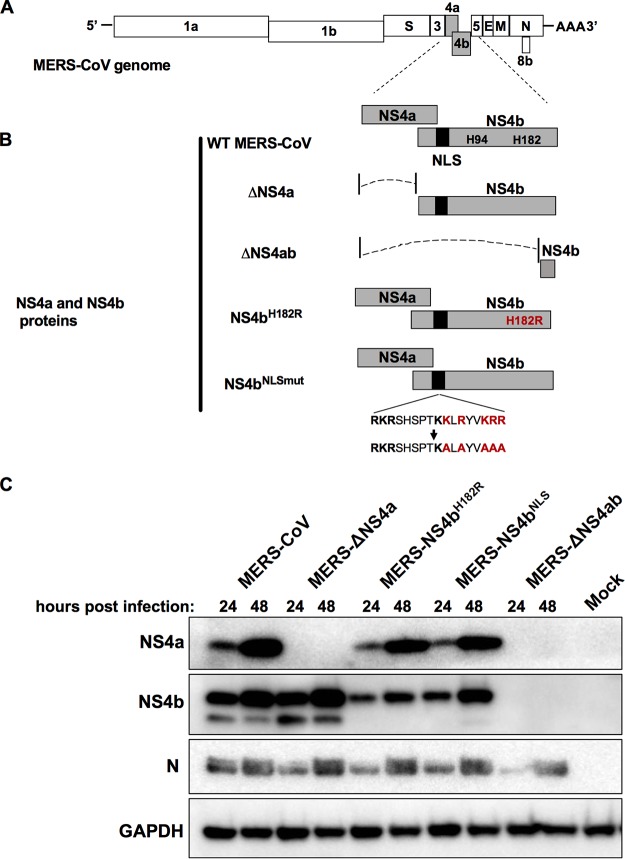
MERS-CoV NS4a and NS4b recombinant mutants. (A) MERS-CoV genome RNA with open reading frames shown. (B) NS4a and NS4b proteins expressed by wild-type and mutant MERS-CoVs. The catalytic His residues of the PDE are shown, and the vertical black bar indicates the NLS of NS4b; the red lettering indicates amino acid substitutions of the catalytic His residue and within the NLS. (C) Expression of viral proteins from recombinant MERS-CoV viruses. A549^DPP4^ cells were infected at an MOI of 10 with WT MERS-CoV, MERS-ΔNS4a, MERS-ΔNS4ab, MERS-NS4b^H182R^, or MERS-NS4b^NLSmut^ or mock infected. Cell lysates were prepared at 24 and 48 h postinfection, analyzed by SDS-PAGE, and probed by Western blotting with rabbit antiserum against NS4a and NS4b or mouse monoclonal antibodies against MERS nucleocapsid protein (N) and GAPDH. The Western blot data are from one representative of three independent infections.

In order to study the effects of NS4a and NS4b on MERS-CoV interactions with the host innate immune system, we used a panel of recombinant MERS-CoV mutants. Deletion mutants MERS-ΔNS4a and MERS-ΔNS4ab were generated from the MERS-CoV infectious clone derived from the MERS-EMC2012 strain (27) as follows and are described in detail in Materials and Methods and diagrammed in Fig. 1A and B. Briefly, MERS-ΔNS4a was generated by altering the start codon (ATG→ATT) and adding an in-frame stop codon 10 codons downstream (TGG→TGA) to ablate synthesis of the NS4a protein. MERS-ΔNS4ab was generated by engineering a 951-nucleotide deletion of open reading frame 4a (ORF4a) and the majority of ORF4b without disrupting the transcription regulatory sequence (TRS) of NS5. To verify the loss of NS4b and/or NS4a expression by these mutants, human A549 cells stably expressing the MERS-CoV receptor DPP4 (A549^DPP4^) were infected with MERS-CoV mutants at a multiplicity of infection (MOI) of 10, and protein lysates were harvested at 24 and 48 h postinfection (hpi) to assess protein expression by Western blotting. As expected, NS4a is not synthesized during infection with MERS-ΔNS4a, and neither protein is detectable during MERS-ΔNS4ab infection (Fig. 1C).

**FIG 2:**
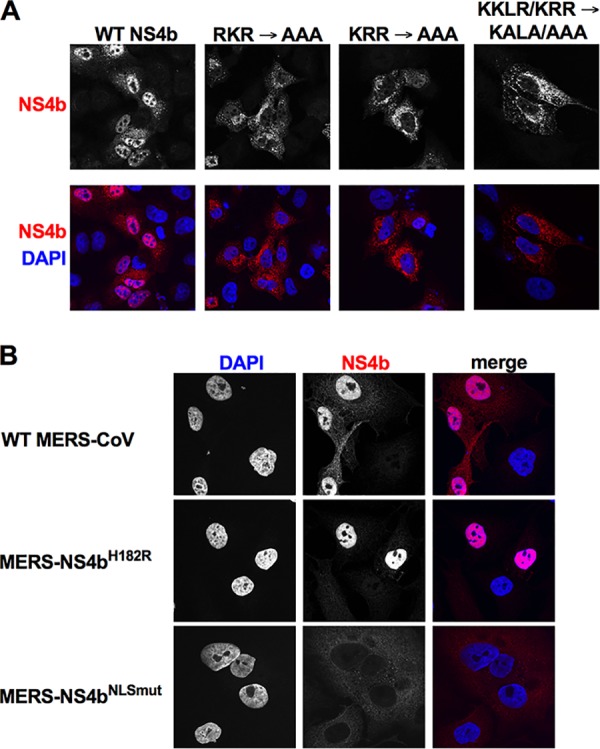
Subcellular localization of MERS-CoV NS4b expression. (A) The nuclear localization signal (NLS) was mapped by mutating basic residues in pCAGGS-NS4b, and NS4b was ectopically expressed in A549 cells by DNA transfection. Twenty-four hours posttransfection, cells were fixed and stained for NS4b using anti-NS4b rabbit serum and goat anti-rabbit AF594 secondary antibody. (B) A549^DPP4^ cells were infected with WT MERS-CoV, MERS-NS4b^H182R^, or MERS-NS4b^NLSmut^ (MOI = 5). Cells were fixed 24 h postinfection and stained with anti-NS4b rabbit serum and goat anti-rabbit AF594 secondary antibody. The images shown in both panels are representative of at least three fields of cells from three independent experiments.

To further investigate the functional domains of NS4b, we utilized two mutant viruses with targeted mutations in either the phosphodiesterase domain or the NLS. MERS-NS4b^H182R^ encodes NS4b with a catalytically inactive phosphodiesterase domain, which was generated from the MERS-CoV infectious clone as previously described (19, 27). The NS4b NLS was previously described as bipartite (RKR_11_KRR), with the first basic motif more potently determining nuclear localization (12, 16). However, this first motif overlaps with the upstream ORF4a, and so mutation of the RKR motif without causing amino acid changes in ORF4a is impossible. To determine how to construct the NS4b NLS mutant (NS4b^NLSmut^), we mapped the nuclear localization signal (NLS) sequence by expressing wild-type (WT) and various NLS mutant NS4b genes from a pCAGGS vector in A549 cells and detecting NS4b proteins by immunofluorescent staining (Fig. 2A). These plasmids expressed NS4b proteins with mutations of the RKR motif, the downstream KRR motif, and a previously undescribed basic motif that lies between the two previously characterized motifs (RKR_5_KKLR_2_KRR). All mutant proteins exhibited primarily cytoplasmic localization; thus, we engineered mutation of the central (KKLR) and downstream (KRR) motifs into the MERS-CoV infectious clone to generate MERS-NS4b^NLSmut^ (Fig. 1B), as described in detail in Materials and Methods (27).

While NS4b expressed during MERS-CoV infection is primarily expressed in the nucleus, during infection with MERS-NS4b^NLSmut^, NS4b exhibits predominantly cytoplasmic localization, as expected (Fig. 2B). During infection with MERS-NS4b^H182R^ and MERS-NS4b^NLSmut^, slightly less NS4b was synthesized than during wild-type (WT) MERS-CoV infection (Fig. 1C), consistent with previous studies of viral PDEs in which expression of mutant protein was less robust than expression of wild-type protein (19). We consistently detected an extra lower band when probing for NS4b. This will be addressed in the Discussion.

### NS4a colocalizes with dsRNA around RTCs.

**FIG 3:**
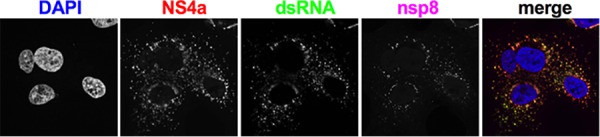
NS4a colocalizes with dsRNA around replication/transcription complexes (RTC) during MERS-CoV infection. A549^DPP4^ cells were infected with WT MERS-CoV (MOI = 5), fixed 24 h postinfection, and stained with rabbit anti-NS4a serum, mouse anti-dsRNA J2, and guinea pig anti-nsp8 serum and then with secondary antibodies goat anti-rabbit AF647, goat anti-mouse AF488, and goat anti-guinea pig AF568. The images shown are representative of at least three fields of cells from three independent experiments.

Previous studies have shown that overexpressed NS4a binds to dsRNA (13, 17). Additionally, NS4a is broadly cytoplasmic when overexpressed in uninfected cells, but colocalizes with dsRNA during infection (11–13). We infected A549^DPP4^ cells with MERS-CoV and used immunofluorescent microscopy to determine NS4a localization. NS4a exhibits a primarily punctate, perinuclear distribution with some diffuse distribution in the cytoplasm (Fig. 3). Cells were costained for NS4a with J2 antibody to detect dsRNA and antiserum against the viral primase, nsp8, a component of the viral polymerase complex and therefore a marker for virus replication/transcription complexes (RTCs) (28). NS4a colocalizes with dsRNA, and both are largely colocalized with nsp8, though dsRNA and NS4a appear more broadly distributed (Fig. 3). This may indicate that either some dsRNA and NS4a localized outside the RTC or the sensitivity of the assay is insufficient to detect all of the nsp8.

### NS4a and NS4b deletion mutants are modestly attenuated in A549^DPP4^ cells.

**FIG 4:**
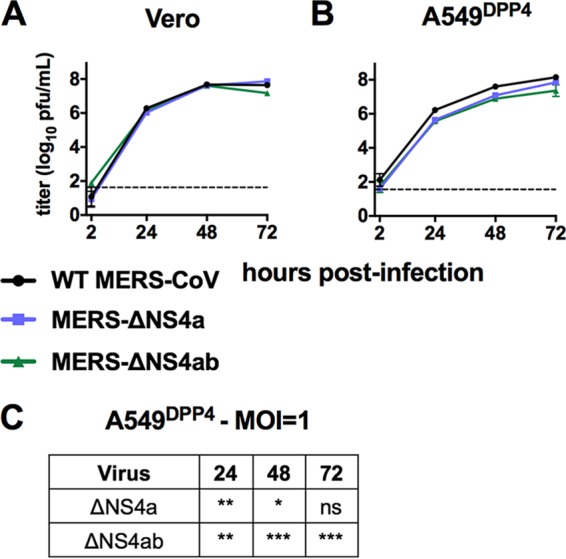
MERS-CoV NS4a and NS4b mutants are attenuated in IFN competent cells. (A) Vero cells were infected in triplicate at an MOI of 1 with WT MERS-CoV, MERS-ΔNS4a, and MERS-ΔNS4ab. Supernatants were collected at indicated times postinfection, and infectious virus was quantified by plaque assay. (B) A549^DPP4^ cells were infected in triplicate at an MOI of 1 with WT MERS-CoV, MERS-ΔNS4a, and MERS-ΔNS4ab, and replication was quantified as in panel A. (C) Statistical significance for mutant virus replication versus WT was calculated by two-way ANOVA. Data are from one representative of three independent experiments. In panel A, the 72-h postinfection data point was only assessed in one out of three experiments. Data are displayed as means ± standard deviation (SD). *, *P* ≤ 0.05; **, *P* ≤ 0.01; ***, *P* ≤ 0.001; ****, *P* ≤ 0.0001.

To assess the impact of NS4a and NS4b mutation on viral replication, we carried out growth curve experiments in Vero and A549^DPP4^ cells with MERS-ΔNS4a and MERS-ΔNS4ab. Vero cells lack a type I IFN response and were used to ensure recombinant viruses are not inherently replication deficient. We infected both cell types with WT or mutant MERS-CoV at an MOI of 1 and harvested supernatant at predetermined times postinfection for titration by plaque assay. All viruses replicated with equivalent kinetics to WT MERS-CoV and to equal titers in Vero cells, indicating that deletion of NS4a and NS4b does not disrupt critical aspects of the viral life cycle (Fig. 4A). In contrast, deletion of NS4a and/or NS4b modestly attenuated MERS-CoV replication in A549^DPP4^ cells at an MOI of 1, with the reductions in titer significant at most time points (Fig. 4B and C). Deletion of both NS4a and NS4b resulted in a slightly greater attenuation than deletion of NS4a alone, though this difference was not statistically significant. That replication of these mutant viruses is attenuated in A549^DPP4^ cells and not in permissive Vero cells strongly suggests that the deficiency is linked to the intact antiviral responses in A549 cells.

### NS4a and NS4b modestly suppress IFN expression.

**FIG 5:**
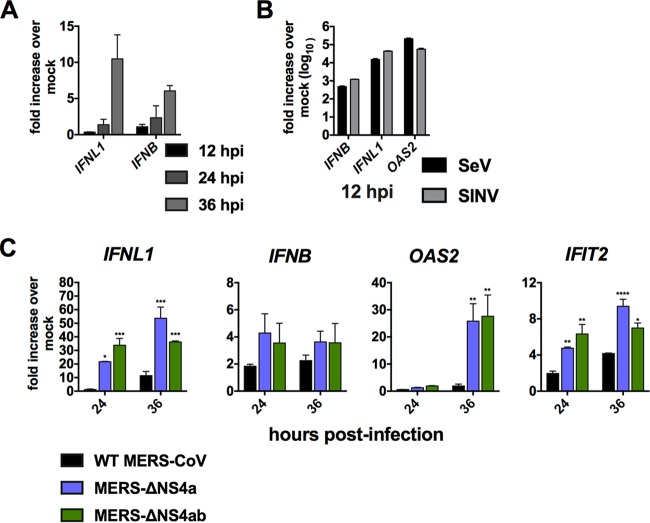
NS4a and NS4b antagonize IFN expression. (A) A549^DPP4^ cells were mock infected or infected in triplicate with WT MERS-CoV at an MOI of 5. RNA was harvested, and gene expression was quantified by qRT-PCR and expressed as fold change over mock infected using the 2^−Δ(Δ^*^CT^*^)^ formula. (B) A549^DPP4^ cells were infected in triplicate with SeV or SINV at an MOI of 5, and at 12 h postinfection, expression of the indicated genes in infected/mock-infected cells was calculated as in panel A. (C) A549^DPP4^ cells were mock infected or infected in triplicate with WT MERS-CoV, MERS-ΔNS4a, and MERS-ΔNS4ab at an MOI of 5 and RNA was harvested at the indicated times postinfection. *IFNL1*, *IFNB*, *OAS2*, and *IFIT2* mRNA levels were quantified by qRT-PCR and calculated over mock-infected cells as in panel A. Data are from one representative of three independent experiments and are displayed as means ± standard errors of the mean (SEM). Statistical significance was calculated by unpaired Student's *t* test: *, *P* ≤ 0.05; **, *P* ≤ 0.01; ***, *P* ≤ 0.001; ****, *P* ≤ 0.0001.

Previous studies of NS4a and NS4b have conflicted on the role of these proteins in suppressing the IFN response (11–14, 15, 18). We aimed to systematically characterize the role of NS4a and NS4b in antagonism of IFN induction during MERS-CoV infection. To ensure that our newly generated A549^DPP4^ cells were a suitable platform for investigating MERS-CoV suppression of the IFN response, we infected them with Sendai virus (SeV), Sindbis virus (SINV), and WT MERS-CoV. In contrast to SeV and SINV, which robustly induced IFN and ISG expression by 12 hpi, MERS-CoV induced little *IFNL1* or *IFNB* expression throughout a 36-h course of infection (Fig. 5A and B).

To determine if NS4a and/or NS4b contributes to suppression of IFN expression, we infected A549^DPP4^ cells with WT MERS-CoV, MERS-ΔNS4a, and MERS-ΔNS4ab and at 24 and 36 h postinfection compared gene expression of IFN and selected ISGs by quantitative real-time PCR (qRT-PCR). In contrast to the minimal increases observed during WT MERS-CoV infection over mock-infected cells, MERS-ΔNS4a or MERS-ΔNS4ab infection resulted in significantly elevated levels of *IFNL1* mRNA and representative ISG *OAS2* and *IFIT2* mRNAs. Interestingly there was no significant induction of type I IFN (Fig. 5C). We did not observe any significant additive effect on antiviral gene expression from the additional deletion of NS4b. However, deletion of ORF4a and/or -b (Fig. 5C) did not result in IFN induction approaching the levels we observed in response to SeV and SINV infection (Fig. 5B), suggesting MERS-CoV encodes additional, potent IFN antagonists and/or utilizes other mechanisms such as sequestration of dsRNA in membrane-bound RTCs to avoid sensing by antiviral receptors.

### NS4b is a novel IFN antagonist.

**FIG 6:**
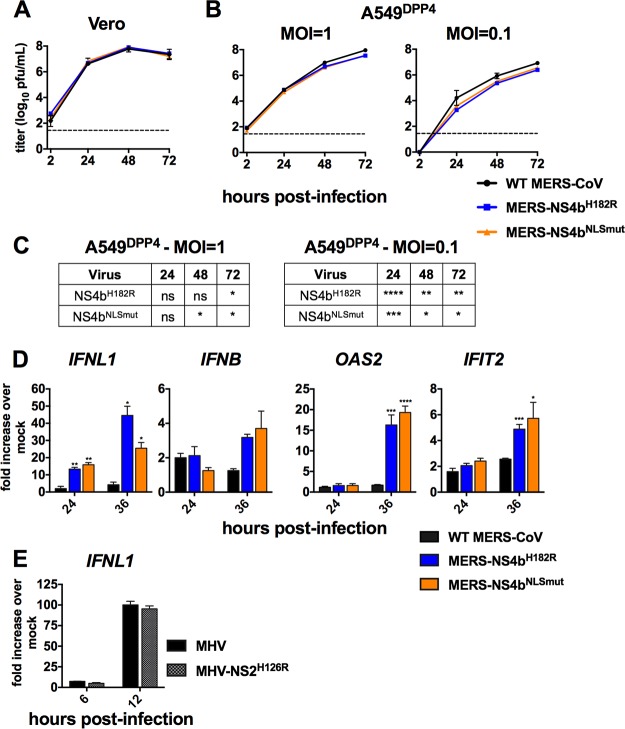
MERS-CoV NS4b NLS and PDE catalytic mutants are attenuated in A549 cells and exhibit increased type III IFN expression. (A) Vero cells were infected in triplicate at an MOI of 1 with WT MERS-CoV, MERS-ΔNS4a, and MERS-ΔNS4ab. Supernatants were collected at indicated times postinfection and infectious virus quantified by plaque assay. (B) A549^DPP4^ cells were infected in triplicate at an MOI of 1 or 0.1 with WT MERS-CoV, MERS-ΔNS4a, and MERS-ΔNS4ab, and replication was quantified as in panel A. Data are from one representative of three independent experiments and are displayed as means ± standard deviation (SD). (C) Statistical significance for mutant virus replication versus WT was determined by two-way ANOVA: *, *P* ≤ 0.05; **, *P* ≤ 0.01, ***, *P* ≤ 0.001; ****, *P* ≤ 0.0001. (D) A549^DPP4^ cells were mock infected or infected in triplicate at an MOI of 5 with WT MERS-CoV, MERS-NS4b^NLS^, and MERS-NS4b^H182R^, and RNA was harvested at the indicated times postinfection. Gene expression over mock-infected cells was measured by RT-qPCR and calculated over mock-infected cells using the 2^−Δ(Δ^*^CT^*^)^ formula. Data are from one representative of three independent experiments and expressed as mean ± SEM. Statistical significance was determined by unpaired Student's *t* test: *, *P* ≤ 0.05; **, *P* ≤ 0.01; ***, *P* ≤ 0.001; ****, *P* ≤ 0.0001. (E) A549^mCEACAM-1^ cells were mock treated or infected with WT MHV or MHV-NS2^H126R^ at an MOI of 5, and RNA was harvested at 6 and 12 h postinfection. *IFNL1* expression was determined as in panel D. Data are from one representative experiment of three.

We previously reported that MERS-CoV NS4b is a member of the 2H-phosphoesterase superfamily of proteins and antagonizes OAS-RNase L activation during MERS-CoV infection through its 2′,5′-PDE activity (19, 29). Unlike previously studied viral PDEs such as mouse hepatitis virus (MHV) NS2, the torovirus pp1a C-terminal domain, and the rotavirus VP3 C-terminal domain, which exhibit primarily cytoplasmic localization (23, 24), NS4b localizes primarily to the nucleus (Fig. 2B), suggesting additional functions. Earlier studies suggested that NS4b nuclear localization might be important for suppressing IFN expression (15), but no previous studies have specifically addressed the role of its catalytic activity in IFN antagonism (30). To characterize the function of the NS4b PDE domain and NLS, we used recombinant MERS-NS4b^H182R^ and MERS-NS4b^NLSmut^. In Vero cells, both mutant viruses replicated with equivalent kinetics to WT MERS-CoV and to equal titers (Fig. 6A). In A549^DPP4^ cells, both viruses are modestly and similarly attenuated at late time points at an MOI of 1, and throughout the course of infection at an MOI of 0.1 where two out of three independent experiments yielded significant differences (Fig. 6B and C). qRT-PCR analysis demonstrated that mutation of either the catalytic site or NLS results in significantly increased IFN and ISG expression during MERS-CoV infection (Fig. 6D).

To further investigate whether PDE-dependent IFN antagonism is unique to MERS-CoV NS4b, we infected A549 cells stably expressing the MHV receptor CEACAM-1 (A549^mCEACAM-1^) with WT MHV or MHV encoding catalytically inactive NS2 (MHV-NS2^H126R^), its native PDE. Both viruses induced slightly more *IFNL1* expression than we observed for MERS-CoV, but MHV-NS2^H126R^ did so to an identical degree as WT MHV (Fig. 6E), demonstrating that the MHV PDE does not antagonize IFN induction in this cell type, consistent with our previous observation in murine cells (31).

**FIG 7:**
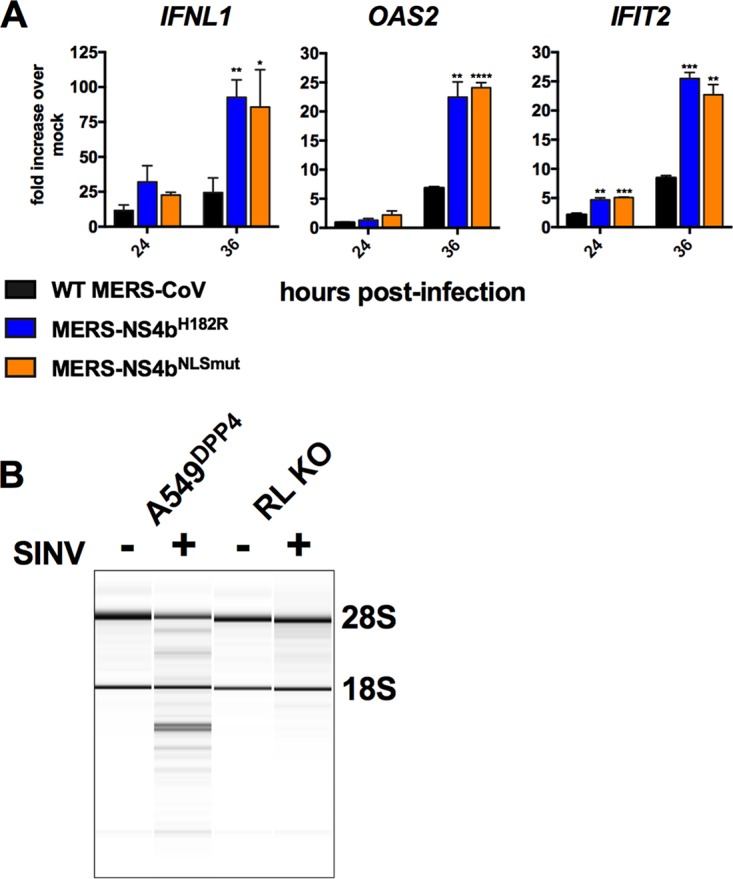
NS4b antagonizes IFN expression independently of RNase L activation. (A) RNase L KO A549^DPP4^ cells were mock infected or infected in triplicate at an MOI of 5 with MERS-CoV, MERS-NS4b^NLS^, and MERS-NS4b^H182R^. RNA was harvested at the indicated times postinfection, mRNA levels expression was quantified by qRT-PCR in and expression in infected/mock-infected cells calculated using the 2^−Δ(Δ^*^CT^*^)^ formula. Data are from one representative experiment of three, expressed as mean ± SEM, and statistical significance was determined by unpaired Student's *t* test: *, *P* ≤ 0.05; **, *P* ≤ 0.01; ***, *P* ≤ 0.001; ****, *P* ≤ 0.0001. (B) A549^DPP4^ and RNase L (RL) KO A549^DPP4^ cells were mock treated or infected with SINV at an MOI of 1 with SINV, and RNA was harvested at 24 h postinfection. RNA was assessed for rRNA degradation using an Agilent Bioanalyzer. The positions of 28S and 18S rRNA are indicated.

Finally, to confirm that NS4b antagonism of IFN expression is a novel viral PDE function and uncoupled from its interaction with the OAS-RNase L pathway, we assessed immune activation by MERS-CoV and NS4b mutants in A549^DPP4^ cells ablated of RNase L expression by CRISPR-Cas9 (i.e., clustered regularly interspaced short palindromic repeats with Cas9) as previously described (32). Both MERS-NS4b^H182R^ and MERS-NS4b^NLSmut^ induced greater *IFNL1*, *OAS2*, and *IFIT2* expression than WT MERS-CoV (Fig. 7A) in RNase L knockout (KO) cells, recapitulating the results we observed in wild-type A549^DPP4^ cells. To confirm that these cells were indeed unable to activate RNase L, cells were infected with SINV, a known potent activator of OAS-RNase L (32), and rRNA integrity was analyzed by Bioanalyzer (Fig. 7B), as previously described (19, 23).

### NS4a does not contribute to OAS-RNase L antagonism during MERS-CoV infection.

**FIG 8:**
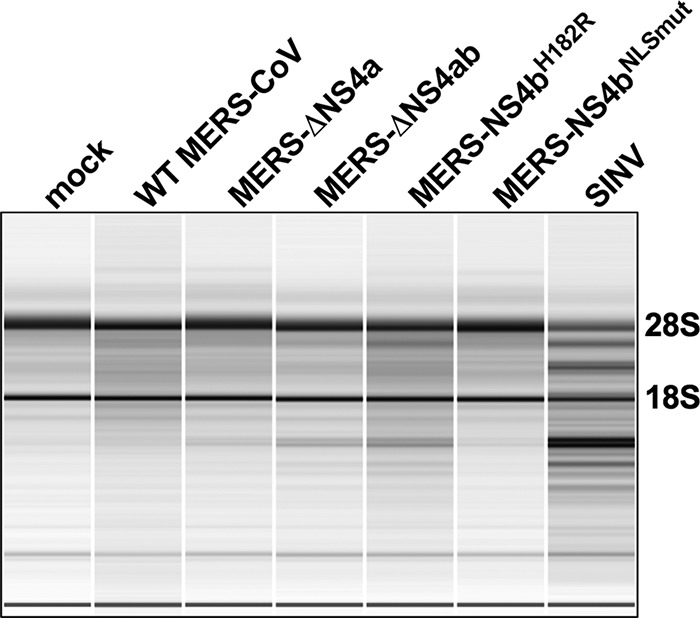
Loss of NS4a does not activate RNase L during MERS-CoV infection. A549^DPP4^ cells were mock infected or infected with WT MERS-CoV, MERS-ΔNS4a, MERS-ΔNS4ab, MERS-NS4b^H182R^, MERS-NS4b^NLSmut^ (MOI = 5), or SINV (MOI = 1). RNA was harvested at 48 h postinfection for MERS-CoV infection and at 24 h postinfection for SINV infection and assessed for rRNA degradation by Agilent Bioanalyzer. 28S and 18S rRNA positions are indicated. Data are from one representative of four independent experiments.

dsRNA binding proteins encoded by viruses such as vaccinia virus (E3L) and influenza A virus (NS1) antagonize activation of the antiviral OAS-RNase L pathway, presumably by sequestration of viral RNA (21, 22, 33). Since RNase L activation by MERS-NS4b^H182R^ is less robust than by other viruses such as SINV in A549^DPP4^ cells (Fig. 7), we hypothesized that NS4a may contribute to antagonism of this pathway during MERS-CoV infection. To test this hypothesis, we infected A549^DPP4^ cells at an MOI of 5, harvested RNA 48 h postinfection, and assessed rRNA degradation using a Bioanalyzer (19, 23). We included SINV as a control for robust RNase L activation (32). RNase L activation is inferred from RNA degradation depicted by the banding pattern in the pseudogel image. MERS-NS4b^H182R^ and MERS-ΔNS4ab induced more rRNA degradation than WT MERS-CoV, indicating activation of RNase L (Fig. 8). Infection with MERS-NS4b^NLSmut^ also did not result in increased rRNA degradation, as expected given previous work demonstrating cytoplasmic PDE localization mediates RNase L antagonism (34). However, infection with MERS-ΔNS4a also did not induce increased rRNA degradation relative to WT MERS-CoV, indicating that the absence of NS4a alone is not enough to activate RNase L in this cell type (Fig. 8). Infection with MERS-ΔNS4ab did not induce more robust rRNA degradation than MERS-NS4b^H182R^, suggesting that NS4a does not play a significant role in antagonism of RNase L during MERS-CoV infection. This result demonstrates that NS4a has both functional similarities to and differences from other viral dsRNA binding proteins.

### NS4a antagonizes PKR activation, but not protein synthesis, during MERS-CoV infection.

**FIG 9:**
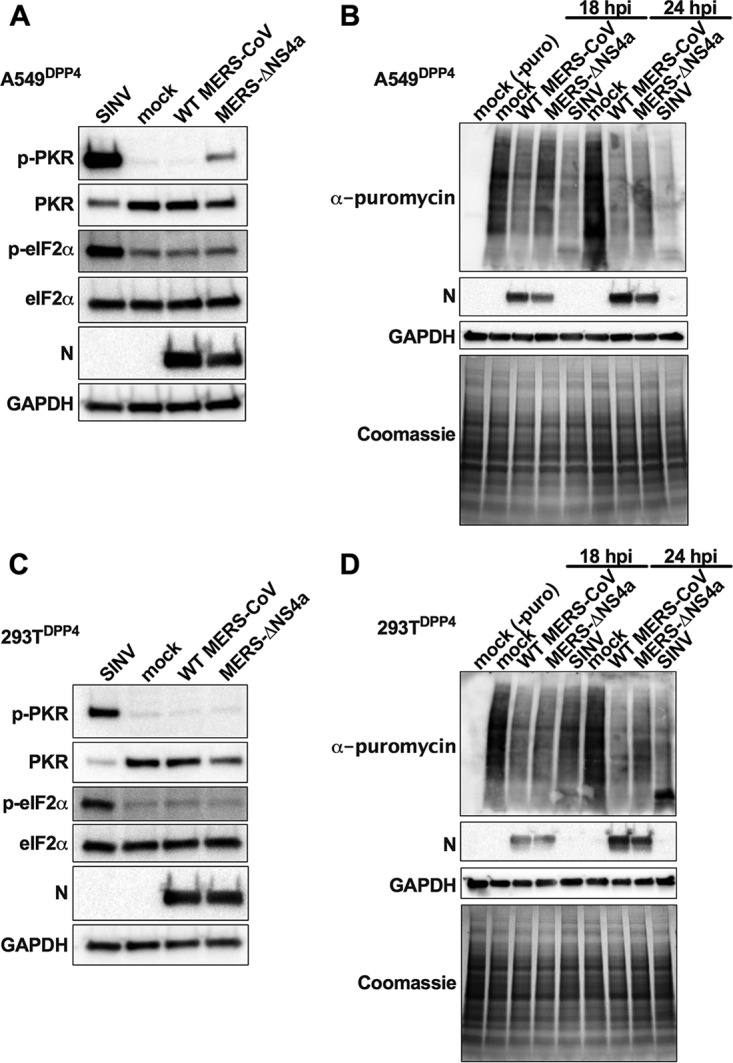
Loss of NS4a activates PKR but does not lead to eIF2α phosphorylation or translation arrest in A549^DPP4^. A549^DPP4^ cells were mock infected or infected with WT MERS-CoV and MERS-ΔNS4a (MOI = 3) or SINV (MOI = 1). (A) Cell lysates were harvested at 24 h postinfection, and proteins were separated by SDS-PAGE and immunoblotted with antibodies against phosphorylated PKR (p-PKR), PKR, phosphorylated eIF2α (p-eIF2α), eIF2α, MERS-CoV N, and GAPDH. (B) Prior to cell lysate harvest, at 18 and 24 h postinfection, cells were treated with puromycin (10 μg/ml) for 10 min. Proteins were separated by SDS-PAGE and analyzed either by immunoblotting with antibodies against puromycin, MERS N protein, or GAPDH or Coomassie stain for labeling of total proteins. (C) 293T^DPP4^ cells were infected and cell lysates harvested as in panel A. (D) 293T^DPP4^ cells were infected and cell lysates harvested as in panel B. Data are from one representative of four (A), three (B), or two (C and D) independent experiments.

A recent study showed that loss of NS4a during infection led to PKR activation, translational arrest, and stress granule formation, but only in certain cell types (18). We investigated whether NS4a antagonizes the dsRNA binding antiviral effector protein kinase R (PKR) during MERS-CoV infection in A549^DPP4^ cells. A549^DPP4^ cells were infected with WT MERS-CoV and MERS-ΔNS4a at an MOI of 3, lysed at 24 h postinfection and analyzed for PKR activation by Western blotting. MERS-ΔNS4a, but not WT MERS-CoV, induced PKR phosphorylation (Fig. 9A). PKR phosphorylation during MERS-ΔNS4a infection was also observed at 16 and 48 h postinfection (data not shown). However, despite the activation of PKR, we did not detect phosphorylation of eIF2α above background levels, suggesting that activation of PKR by MERS-ΔNS4a in A549^DPP4^ cells is not sufficient to engage downstream elements of this pathway or that MERS-CoV encodes an additional antagonist that blocks steps downstream of PKR phosphorylation. In contrast, SINV infection promotes robust phosphorylation of PKR and eIF2α in the same cells, indicating the lack of eIF2α phosphorylation during MERS-ΔNS4a is not due to a deficiency of this pathway in A549^DPP4^ cells (Fig. 9A).

Although we did not detect eIF2α phosphorylation by immunoblotting, we wanted to confirm that PKR activation during MERS-ΔNS4a infection does not mediate translation arrest in A549^DPP4^ cells. Thus, we compared protein synthesis during infection with MERS-ΔNS4a and WT MERS-CoV. We either mock infected or infected A549^DPP4^ cells with WT MERS-CoV or MERS-ΔNS4a. We treated cells 18 and 24 h postinfection with puromycin for 10 min to label nascent proteins prior to protein harvest. We used immunoblotting with an antipuromycin antibody to specifically detect newly synthesized proteins and used Coomassie staining to assess total protein levels (35). Decrease in puromycin signal indicates translation arrest. Puromycin signal was not lower in MERS-ΔNS4a-infected A549^DPP4^ cells compared to WT MERS-CoV, indicating PKR phosphorylation did not induce downstream translation arrest (Fig. 9B).

In contrast to A549^DPP4^ cells, we observe no phosphorylation of PKR during MERS-ΔNS4a infection in 293T^DPP4^ cells (Fig. 9C). Furthermore, MERS-CoV shut down protein synthesis during infection of these cells as previously reported with no enhancement of translation arrest from deletion of NS4a (36) (Fig. 9D). This confirms the observed loss of protein synthesis occurs by an NS4a-independent mechanism and highlights that differences in cell type may affect levels of activation of the dsRNA-induced innate immune pathways.

## DISCUSSION

Studies from other labs as well as data presented herein have demonstrated that MERS-CoV only modestly induces three major antiviral pathways: IFN production and signaling, OAS-RNase L, and PKR. This is likely due largely to viral antagonists of dsRNA-induced host responses. Our study, as well as recent reports from other labs, has shown that deletion of MERS-CoV accessory proteins from recombinant viruses leads to enhanced activation of antiviral pathways. However, these effects are relatively small compared to those in other RNA viruses, and deletion of these accessory proteins only mildly attenuates replication. This is in contrast to early studies utilizing overexpression and reporter plasmids or ectopic expression from heterologous virus showing robust suppression of *IFNB* induction by NS4a and NS4b (11, 13–16). Thus, caution is warranted in extrapolating from studies that rely only on ectopic expression.

We have used recombinant MERS-CoV mutants to study interactions between the accessory proteins NS4a and NS4b and the host immune response. All of the viruses with mutations or deletions in NS4a and NS4b were modestly attenuated compared to WT MERS-CoV in A549^DPP4^ cells. These modest differences are consistent with previous studies of MERS-CoV accessory proteins (10, 12, 14, 18, 30). Furthermore, there is a clinical report of human isolates with a 16-amino-acid deletion in NS4a (37) and West African camel MERS-CoV isolates with ORF3 and ORF4b deletions, likely due to founder effects upon introduction into these populations (30). The isolation of these viruses supports findings that MERS-CoV accessory proteins are not definitive determinants of viral replication. However, all other known circulating MERS-CoV isolates and MERS-CoV-like viruses carry intact accessory ORFs, strongly suggesting that these proteins do play important roles in promoting viral fitness.

We found roles for both NS4a and NS4b in suppressing *IFNL1* expression in response to MERS-CoV infection, which is notably muted compared to that in other RNA viruses (Fig. 5 and 6). The lack of a similar increase in *IFNB* expression in response to mutant MERS-CoV infection is likely due to generally less robust expression of *IFNB* in A549 cells, which preferentially express *IFNL1* like other epithelial cells derived from barrier surfaces (38). We found that NS4b IFN antagonism was dependent on nuclear localization, confirming an earlier report (15), and its catalytic activity.

NS4b is the first viral phosphodiesterase known to suppress antiviral pathways in addition to RNase L, distinguishing it from phosphodiesterases found in the genomes of other coronavirus subgenera (Fig. 6). While the exact mechanism of NS4b IFN antagonism remains unclear, several host-encoded PDEs within the same protein family are known or believed to participate in various steps of RNA processing (29, 39). Whether, like some cellular PDEs (40), NS4b can cleave 3′-5′-linked phosphodiester bonds in addition to 2′-5′ oligoadenylates and whether it mediates any of its immune antagonist functions through directly or indirectly acting on host RNAs is an ongoing area of study. Finally, our data demonstrate that NS4b antagonism of IFN is distinct from its RNase L antagonist activity (Fig. 7), demonstrating that NS4b has at least two independent functions.

We observed reduced expression of mutant NS4b compared to WT protein, as we reported previously (19). It is not known whether this reduced expression is due to reduced protein stability or to the antibody not recognizing the mutant protein as efficiently as the WT protein. However, the abundance of NS4b during infection with MERS-NS4b^NLSmut^, though lower than that of WT protein, is sufficient to fully prevent RNase L activation, indicating mutation does not reduce NS4b levels below an effective concentration (23, 34, 41) (Fig. 7). Thus, it is unlikely that decreased mutant protein abundance is responsible for the observed IFN phenotype (24). We observed a faster-migrating protein, also staining with antiserum directed against NS4b (Fig. 1C). We presume that this faster-migrating protein was not detected in NS4b mutant-infected cells due to its lower expression level relative to full-length NS4b and because it is weakly expressed even in WT MERS-CoV NS4b cells. We do not know the identity of this band. However, we speculate it could be a breakdown product of full-length NS4b or more interestingly a protein initiated at one of several ATGs located downstream and in frame with the NS4b initiation site.

Activation of RNase L during MERS-NS4b^H182R^ infection is less robust than during infection with MHV-NS2^H126R^ in macrophages (23) or SINV infection of A549 cells (32) (Fig. 7), suggesting MERS-CoV may have redundant mechanisms for inhibiting this pathway. Based on the role of the viral dsRNA binding proteins NS1 of influenza virus and E3L of vaccinia virus (21, 22, 33) in blocking RNase L activation as well as IFN and PKR, we hypothesized that NS4a contributes to antagonism of OAS-RNase L. Surprisingly, infection with MERS-ΔNS4a did not induce increased rRNA degradation compared to wild-type virus, nor did NS4a deletion produce any additive effect on RNase L activation in combination with deletion of NS4b. Nevertheless, the lack of robust RNase L activation even when NS4b is catalytically inactive suggests the possibility MERS-CoV does encode additional antagonists. One intriguing possibility is nsp15; its MHV ortholog has recently been described as contributing to evasion of multiple dsRNA-sensing pathways (42, 43). Alternatively, as has been speculated for MHV, MERS-CoV dsRNA may be contained, even in the absence of NS4a, in viral replication/transcription complexes (RTCs) and therefore hidden from antiviral sensors (44, 45).

Due to its dsRNA-binding activity, we also hypothesized that NS4a inhibits PKR activation. One previous study showed that ectopically expressed NS4a inhibits PKR activation and can functionally replace the native PKR antagonist of encephalomyocarditis virus (14). Deletion of NS4a within recombinant MERS-CoV has previously been shown to result in enhanced translation arrest compared to WT MERS-CoV in HeLa cells (18). Consistent with this, we found that deletion of NS4a results in PKR phosphorylation, but in A549^DPP4^ cells, this did not lead to phosphorylation of eIF2α above background levels, and MERS-ΔNS4a did not induce more translation arrest than WT MERS-CoV. In 293T^DPP4^ cells, MERS-CoV induced translation arrest as previously reported (36), but we did not observe a more robust effect during MERS-ΔNS4a infection. Furthermore, PKR was not phosphorylated in 293T^DPP4^ cells during MERS-ΔNS4a infection, confirming the PKR-independent mechanism of translational arrest and highlighting differences between cell types in antiviral pathway activation. These differences demonstrate the importance of using multiple model systems to fully elucidate interactions between viral proteins and host immune pathways.

Despite the lack of robust replication phenotypes, studies of MERS-CoV accessory proteins from other labs as well as our own have identified novel and important virus-host interactions that likely contribute in important ways to maintenance of MERS-CoV in its ecological niche and possibly during infection of the human respiratory tract. Future work on MERS-CoV accessory proteins in animal models and *in vitro* systems that more faithfully recapitulate the human airway should more fully answer the question of how these proteins contribute to replication under immune pressure and to pathogenesis.

## MATERIALS AND METHODS

### Recombinant viruses.

Recombinant WT MERS-CoV and mutants were derived from the EMC/2012 strain cDNA clone, all by introducing mutations into cDNA fragment F assembling the genome fragment and recovering infectious virus as described previously (27).

To ablate expression of MERS NS4a, PCR was performed with primers EMCmut4A (5′-NNNNNNTTAATTAACGAACTCTATTGATTACGTGTCTCTGCTTAATCAAATTTGACAGAAGTACCTTAACTC-3′) and MERS:F3941 (5′-CACCGAAATGCATGCCAGCC-3′). The positions of the F3941 within the MERS genome are 28321 to 28302. This product was digested with PacI and NcoI, gel purified, and then ligated into the MERS F plasmid, which had been similarly digested.

To remove MERS NS4a and NS4b expression, PCR was performed with primers delta4AB (5′-NNNNNNTTAATTAAGTTCATTCTTATCCCATTTTACATC-3′) and MERS:F3415 (5′-GAGGGGGTTTACTATCCTGG-3′). This product was digested with PacI and SanDI, gel purified, and then ligated into the MERS F plasmid, which had been similarly digested. The delta4AB primer uses the PacI site just upstream of NS4a, and then the rest of this primer’s sequence is from positions 26795 to 26819 in the MERS genome. The deletion removes nucleotides 25844 to 26794 in the MERS genome and does not disrupt either the ∼40 nucleotides upstream of or the transcription regulatory sequence (TRS) of NS5.

MERS-NS4b^H182R^ was previously described (19). MERS-4b^NLSmut^ was constructed by substituting alanine for each of residues 31, 33, 36, 37, 38, and 43. Briefly, one PCR product was generated using primers MERS:F1376 (5′-GTTTCTGTCGATCTTGAGTC-3′) and MERS4bR (5′-NNNNNNCGTCTCGCAACGTAGGCCAGTGCCTTAGTTGGAGAATGGCTCCTC-3′). A second PCR was performed with the primers MERS4bF (5′-NNNNNNCGTCTCCGTTGCGGCTGCATTTTCTCTTCTGGCCCATGAAGACCTTAGTGTTATTG-3′) and MERS:F3415 (5′-GAGGGGGTTTACTATCCTGG-3′). The positions of the F1376 primer in the context of the MERS genome are 25748 to 25767, while the positions for the reverse F3415 primer are 27815 to 27796. The products were gel isolated, digested with BsmBI (underlined in the primers shown above), and ligated with T4 DNA ligase. The resultant product was digested with PacI and SanDI, gel purified, and then used to replace the corresponding region in the MERS F plasmid which had been similarly digested. All recombinant viruses were isolated as previously described (27).

Sindbis virus Girdwood (G100) (SINV) was obtained from Mark Heise, University of North Carolina, Chapel Hill, and prepared as previously described (46), and Sendai virus (SeV) Cantell strain was obtained from Carolina Lopez, University of Pennsylvania, Philadelphia, and prepared as previously described (47).

### Cell lines.

Vero CCL-81 cells were cultured in Dulbecco’s modified Eagle’s medium (DMEM) plus 10% fetal bovine serum (FBS), penicillin-streptomycin, gentamicin, sodium pyruvate, and HEPES. Human A549 cells were cultured in RPMI 1640 supplemented with 10% FBS and penicillin-streptomycin. A549^DPP4^ and 293T^DPP4^ cells were constructed by lentivirus transduction of *DPP4*. The plasmid encoding the cDNA of *DPP4* was purchased from Sino Biological. The cDNA was amplified using forward primer 5′-GACTCTAGAATGAAGACACCGTGGAAGGTTCTTC-3′ and reverse primer 5′-TCGAGACCGAGGAGAGGGTTAGGGATAGGCTTACCAGGTAAAGAGAAACATTGTTTTATG-3′. A V5 tag was introduced to the 3′ end of the cDNA by PCR to enable easy detection of DPP4. The amplicon was cloned into pCR4-TOPO TA cloning vector (Invitrogen K457502), to make pCR4-DDP4-V5. The fragment containing DPP4-V5 was digested by the XbaI and SalI restriction enzymes from pCR4-DPP4-V5 and was cloned into pLenti-GFP in place of green fluorescent protein (GFP), generating pLenti-DPP4-V5. The resulting plasmids were packaged in lentiviruses pseudotyped with vesicular stomatitis virus glycoprotein G (VSV-G) to establish the gene knock-in cells as previously described (32). Forty-eight hours after transduction, cells were subjected to hygromycin (1 mg/ml) selection for 3 days and single-cell cloned. Clones were screened for DPP4 expression and susceptibility to MERS-CoV replication. RNase L knockout A549^DPP4^ cells were generated as previously described for parental A549 cells (32). A549^mCEACAM-1^ cells were generated as described above for A549^DPP4^ cells, but by insertion of mouse *Ceacam-1* (GenBank accession no. NM_001039185.1) into the lentivirus vector rather than human *DPP4*.

### NS4b expression from pCAGGS plasmid.

WT NS4b and the indicated mutant NS4b constructs were synthesized and purchased from Bio Basic in vector pUC57 flanked by restriction sites ClaI and XhoI. pUC57 plasmids were digested and NS4b fragments gel purified for ligation into pCAGGS expression vector. Ectopic expression was conducted using Lipofectamine 2000 transfection reagent (Thermo Fisher no. 11668027) following the provided protocol. At 24 h posttransfection, cells were fixed and stained as described below.

### MERS-CoV infections and titration.

Viruses were diluted in serum-free RPMI and added to cells for absorption for 45 min at 37°C. Cells were washed three times with phosphate-buffered saline (PBS) and fed with RPMI plus 2% FBS. One hundred fifty microliters of supernatant was collected at the times indicated and stored at −80°C for titration by plaque assay on Vero CCL-81 cells as previously described (27). All infections and virus manipulations were conducted in a biosafety level 3 (BSL3) laboratory using appropriate personal protective equipment and protocols.

### Immunofluorescent staining.

At indicated times postinfection, cells were fixed with 4% paraformaldehyde for 30 min at room temperature. Cells were then washed three times with PBS and permeabilized for 10 min with PBS plus 0.1% Triton X-100. Cells were then blocked in PBS and 2% bovine serum albumin (BSA) for 45 to 60 min at room temperature. Primary antibodies were diluted in blocking buffer and incubated on a rocker at room temperature for 1 h. Cells were washed three times with blocking buffer and then incubated with rocking at room temperature for 30 min with secondary antibodies diluted in blocking buffer. Finally, cells were washed twice with blocking buffer and once with PBS, and nuclei were stained with DAPI (4′,6-diamidino-2-phenylindole) diluted in PBS. Coverslips were mounted onto slides for analysis by confocal microscopy. NS4b was detected using anti-NS4b rabbit serum at 1:500 and NS4a with anti-NS4a rabbit serum at 1:500 (both obtained from Luis Enjuanes, Spanish National Centre for Biotechnology) (12). dsRNA was detected using commercial antibody J2 at 1:1,000 and nsp8 using anti-nsp8 guinea pig serum (obtained from Mark Denison, Vanderbilt University). Secondary antibodies were all highly cross-adsorbed IgG (H+L) from Invitrogen: goat-anti rabbit AF594 (catalog no. AA11037), goat anti-mouse AF488 (catalog no. AA11029), goat anti-rabbit AF647 (catalog no. A32733), goat anti-guinea pig AF594 (catalog no. A11076), and goat anti-guinea pig AF568 (catalog no. A11075).

### Western immunoblotting.

Cells were washed twice with ice-cold PBS and lysates harvested at indicated times postinfection with lysis buffer (1% NP-40, 2 mM EDTA, 10% glycerol, 150 mM NaCl, 50 mM Tris HCl) supplemented with protease inhibitors (Roche cOmplete mini-EDTA-free protease inhibitor) and phosphatase inhibitors (Roche PhosStop Easy Pack). After 5 min, lysates were harvested, incubated on ice for 20 min, and centrifuged for 20 min at 4°C, and supernatants were mixed 3:1 with 4× Laemmli sample buffer. Samples were heated at 95°C for 5 min and then separated by 4 to 15% SDS-PAGE and transferred to polyvinylidene difluoride (PVDF) membranes. Blots were blocked with 5% nonfat milk and probed with the following antibodies diluted in the same blocking buffer: anti-PKR (phospho-T446 [E120]) rabbit monoclonal antibody (MAb) at 1:1,000 (Abcam 32036), anti-PKR (D7F7) rabbit MAb at 1:1,000 (Cell Signaling Technology no. 12297), anti-GAPDH (anti-glyceraldehyde-3-phosphate dehydrogenase [14C10]) rabbit MAb (Cell Signaling Technology no. 2118) at 1:1,000, SinoBiological anti-MERS N mouse MAb at 1:1,000, anti-NS4a rabbit serum at 1:500 (obtained from Luis Enjuanes, Spanish National Centre for Biotechnology) (12), and anti-NS4b rabbit serum at 1:500 (obtained from Robert Silverman, Cleveland Clinic) (12). For detection of eIF2α and phosphorylated eIF2α, blots were blocked with 5% BSA and probed with phospho-eIF2α (Ser51) antibody diluted in blocking buffer at 1:1,000 (Cell Signaling Technology no. 9721). The secondary antibodies used were horseradish peroxidase (HRP)-conjugated Santa Cruz goat anti-mouse IgG secondary antibody (SC2005) at 1:5,000 and HRP-linked Cell Signaling Technology anti-rabbit IgG secondary antibody (CS7074) at 1:3,000. Blots were visualized using Thermo Scientific SuperSignal West chemiluminescent substrates (catalog no. 34095 or 34080). Blots were probed sequentially with antibodies and between antibody treatments were stripped using Thermo Scientific Restore Western blot stripping buffer (catalog no. 21059).

Protein synthesis was assessed by treatment of cells with 10 μg/ml puromycin for 10 min prior to protein harvest (35). Lysates were harvested and run on SDS-PAGE gels as described above. For detection of puromycin, antipuromycin mouse MAb (Millipore clone 4G11 MABE342) was used at 1:6,000, and the secondary antibody used was goat anti-mouse IgG-HRP (Thermo Scientific no. 31430) at 1:3,000. For detection of total protein by Coomassie staining, cell lysates (as prepared above) were separated by 4 to 15% SDS-PAGE. Gels were fixed and stained with 0.05% Coomassie brilliant blue R250 (Bio-Rad no. 161-0400) in 50% methanol–10% acetic acid solution for 2 h with gentle rocking at room temperature. Gels were destained with 7% methanol and 5% acetic acid for several hours and then imaged.

### qRT-PCR.

At indicated times postinfection, cells were lysed with buffer RLT Plus (Qiagen RNeasy Plus no. 74136) and RNA extracted following the prescribed protocol. cDNA was synthesized according to the protocol for Thermo Scientific Superscript III reverse transcriptase (Thermo Scientific no. 18080044). RT-qPCR was performed under conditions validated for the indicated primer set. The forward (F) and reverse (R) primer sequences are as follows: *IFNL1*, F, 5′-CGCCTTGGAAGAGTCACTCA-3′, and R, 5′-GAAGCCTCAGGTCCCAATTC-3′; *OAS2*, F, 5′-TTCTGCCTGCACCACTCTTCACGAC-3′, and R, 5′-GCCAGTCTTCAGAGCTGTGCCTTTG-3′; *IFIT2*, F, 5′-CTGAGAATTGCACTGCAACCATG-3′, and R, 5′-TCCCTCCATCAAGTTCCAGGTGAA-3′; *IFNB*, F, 5′-GTCAGAGTGGAAATCCTAAG-3′, and R, 5′-ACAGCATCTGCTGGTTGAAG-3′; and *GAPDH*, F, 5′-GCAAATTCCATGGCACCGT-3′, and R, 5′-TCGCCCCACTTGATTTTGG-3′. Fold changes in mRNA were calculated using the threshold cycle (*C_T_*) formula 2^−Δ(Δ^*^CT^*^)^ (Δ*C_T_* = *C_T_*_gene of interest_ − *C_T_*_GAPDH_) and expressed as fold infected/mock infected.

### Analyses of RNase L-mediated rRNA degradation.

RNA was harvested with buffer RLT (Qiagen RNeasy no. 74106) and analyzed on an RNA chip with an Agilent Bioanalyzer using the Agilent RNA 6000 Nano kit and its prescribed protocol, as we have described previously (catalog no. 5067-1511) (19, 23).

### Statistical analysis.

Plotting of data and statistical analysis were performed using GraphPad Prism software (GraphPad Software, Inc.). Statistical significance was determined by two-way analysis of variance (ANOVA) for viral replication curves and by unpaired Student’s *t* test for RT-qPCR.
